# SVA Retrotransposons and a Low Copy Repeat in Humans and Great Apes: A Mobile Connection

**DOI:** 10.1093/molbev/msac103

**Published:** 2022-05-16

**Authors:** Annette Damert

**Affiliations:** Infection Biology Unit and Primate Genetics Laboratory, German Primate Center, Leibniz Institute for Primate Research, Göttingen, Germany

**Keywords:** segmental duplication, retrotransposon, lcr16a, SVA

## Abstract

Segmental duplications (SDs) constitute a considerable fraction of primate genomes. They contribute to genetic variation and provide raw material for evolution. Groups of SDs are characterized by the presence of shared core duplicons. One of these core duplicons, low copy repeat (lcr)16a, has been shown to be particularly active in the propagation of interspersed SDs in primates. The underlying mechanisms are, however, only partially understood. *Alu* short interspersed elements (SINEs) are frequently found at breakpoints and have been implicated in the expansion of SDs.

Detailed analysis of lcr16a-containing SDs shows that the hominid-specific SVA (SINE-R-VNTR-*Alu*) retrotransposon is an integral component of the core duplicon in Asian and African great apes. In orang-utan, it provides breakpoints and contributes to both interchromosomal and intrachromosomal lcr16a mobility by inter-element recombination. Furthermore, the data suggest that in hominines (human, chimpanzee, gorilla) SVA recombination-mediated integration of a circular intermediate is the founding event of a lineage-specific lcr16a expansion. One of the hominine lcr16a copies displays large flanking direct repeats, a structural feature shared by other SDs in the human genome.

Taken together, the results obtained extend the range of SVAs’ contribution to genome evolution from RNA-mediated transduction to DNA-based recombination. In addition, they provide further support for a role of circular intermediates in SD mobilization.

## Introduction

Segmental duplications (SDs) or low copy repeats (lcrs) are large (>1 kb) duplicated segments of genomic DNA typically more than 90% identical in sequence (Bailey et al. 2001). Groups of SDs are characterized by the presence of core duplicons around which duplication blocks are organized (Marques-Bonet and Eichler 2009). The best-investigated core duplicon in primates is lcr16a, a 20 kb segment present in several copies on human chromosome 16 (Loftus et al. 1999). In the genome, lcr16a is mostly found associated with other SDs in large duplication blocks (Johnson et al. 2006; Cantsilieris et al. 2020). A gene family (*morpheus*; *NPIP*—nuclear pore complex interacting protein) subject to strong positive selection has been identified within lcr16a duplication blocks (Johnson et al. 2001). Lcr16a exists in a single copy in Old World monkeys (OWM—macaque, baboon). It amplified independently in the orang-utan and hominine (gorilla, chimpanzee, human) lineages, respectively (Johnson et al. 2006). Among these, orang-utans are the only ones with a substantial number of copies on a single chromosome outside of chromosome 16. In orang-utan and OWMs, only the ancestral NPIPA protein is encoded. In hominines, the lcr16a expansion led to the emergence and amplification of *NPIPB*—a variant characterized by an alternative first exon and different patterns of alternative splicing (Bekpen et al. 2017; Cantsilieris et al. 2020).

During their first characterization of lcr16a, Johnson et al. (2006) observed a 3-fold enrichment of *Alu* elements at the boundaries of lcr16a-associated SDs. *Alu*-mediated mechanisms have been suggested to play a role in the generation of copy number variants and SDs (Bailey et al. 2003; Han et al. 2007; Boone et al. 2014). *Alu*s are the most abundant interspersed repeats in primate genomes (Batzer and Deininger 2002). In the human genome, there are around 1 million copies (Lander et al. 2001). The hominoid-specific SVA (**S**INE-R-**V**NTR-***A****lu*) elements are present in gibbons; amplification to higher copy numbers, however, occurred in great apes only (Wang et al. 2005). SVA expansion in orang-utan has been independent from that in the hominine lineage (Lupan et al. 2015; Damert 2018).

SVA retrotransposons have been shown to contribute to genome evolution by co-mobilizing sequences at both their 5′ (Damert et al. 2009; Hancks et al. 2009) and 3′ ends (Xing et al. 2006) in the process of RNA-mediated transposition (referred to as 5′ and 3′ transduction, respectively). Reports on the involvement of SVAs in genomic rearrangements at DNA level are scarce and limited to recombination-mediated deletions (Lee et al. 2012; Kherraf et al. 2018).

Here I report that SVA elements contribute to the expansion of lcr16a in great apes: in orang-utan an SVA forms an integral component of the core duplicon and mediates its interchromosomal and intrachromosomal spread. In hominines, a subgroup of lcr16a copies can be traced back to a duplication event involving a circular intermediate and SVA–SVA recombination.

## Results

### SVA is an Integral Component of the lcr16a Core Duplicon in Orang-utan

**Fig. 1.**
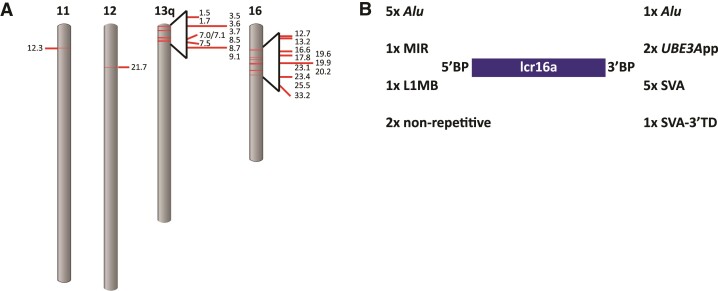
Distribution of lcr16a duplications in the orang-utan genome and characteristics of their breakpoints. (*A*) The positions of the lcr16a duplicates on Susie_PAB_pri (Vollger et al. 2022) are mapped against the ponAbe3 ideogram available at the NCBI genome decoration page. (*B*) The number of repetitive elements overlapping the independent breakpoints (BP) at the lcr16a 5′ and 3′ end, respectively. *UBE3A*pp, *UBE3A* processed pseudogene; TD, transduction.

In the course of an analysis of SVAs in the orang-utan genome, I noticed a group of nearly identical elements characterized by a common 3′ transduction and similar genomic context at their 3′ ends. Further analysis revealed that they are associated with the lcr16a core duplicon. In the ponAbe3 reference assembly, 11 lcr16a copies could be identified. Half of them mapped to sequences without definitive chromosome assignment (chrUn or random); copies mapped to sequences with definitive chromosomal assignment were found to be associated with assembly gaps. To obtain a more comprehensive picture, lcr16a-containing BAC sequences (Cantsilieris et al. 2020 and additional sequences retrieved from the NCBI database) were analyzed. This way, a total of 33 different lcr16a copies (ppy_1–33) were identified (supplementary table S1, Supplementary Material online; note that with the similarity between copies approaching that of allelic variation, in some cases it cannot be decided whether they represent independent copies or alleles of the same copy). Once it became available, the copies were mapped against the new orang-utan high-fidelity (HiFi) assembly (Susie_PAB_pri, Vollger et al. 2022). Out of the 33 lcr16a-containing duplications 21 could be mapped (fig. 1*A*). In addition, three copies not represented on BACs (ppy_10a, 16a, and 20a) were found in the HiFi assembly.

Analysis of the junctions linking lcr16a with co-mobilized SDs or unique sequence identified nine independent breakpoints at the 5′ and nine independent breakpoints at the 3′ end of the lcr16a core duplicon (fig. 1*B*, supplementary tables S2 and S3, Supplementary Material online). Only two of the breakpoints were found to localize to nonrepetitive sequence; the remainder overlaps with retrotransposons and, in two instances, with a processed pseudogene. Strikingly, five out of the nine lcr16a 3′ breakpoints localize to the SVA sequence and one to the SVA-associated 3′ transduction observed in the initial analysis. Among the other junctions linking either lcr16a-associated SDs with each other or with unique sequence at the insertion site only a single instance of an SVA element overlapping a boundary could be identified (transition between chromosome 10 and chromosome 13 sequences in ppy_20a). *Alu* elements, by contrast, were found at 9 out of 20 of these junctions. Thus, considering that lcr16a mostly moves in the context of larger duplication blocks, in which associated SDs provide the insertion breakpoints, the contribution of SVA to lcr16a mobility is limited.

**Fig. 2.**
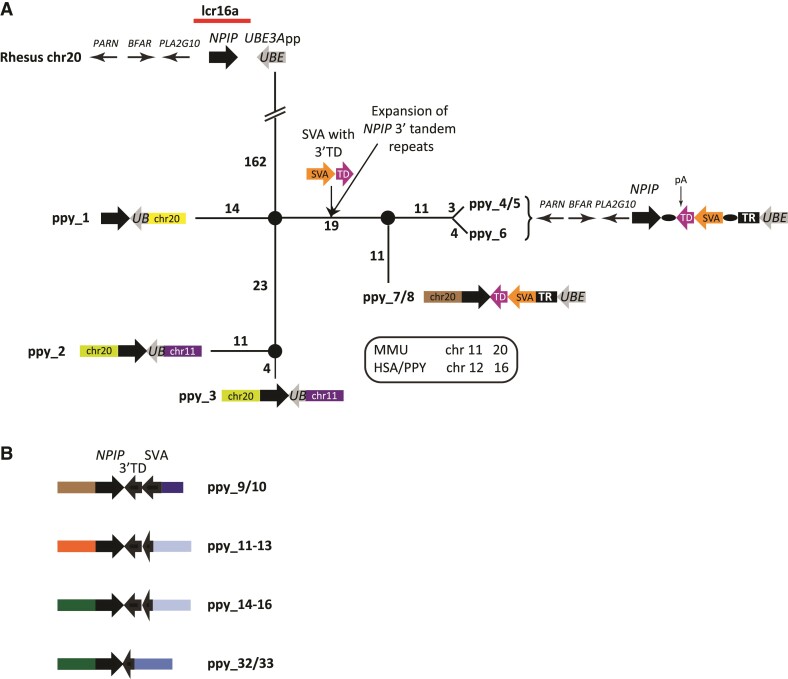
SVA is an integral component of lcr16a in orang-utan. (*A*) Median-joining network of orang-utan lcr16a copies containing the *UBE3A* processed pseudogene. The network is rooted on the rhesus macaque *UBE3A* processed pseudogene (*UBE3A*pp) which is shown in the context of the rhesus ancestral lcr16a locus on top of the network. Except for the path connecting the root and the first node the network is drawn to scale. The number of substitutions is indicated for each path. In ppy_1–3, the lcr16a 3′ breakpoint localizes to *UBE3A*pp (*UBE*—gray arrows). Copies ppy_4–8 are characterized by the insertion of an SVA carrying a 3′ TD into the expanded *NPIP* 3′ tandem repeats. TSDs (black ovals) are discernible in ppy_4–6 which, based on the order of the upstream genes, represent the orang-utan lcr16a ancestral locus. TR—tandem repeats downstream of the SVA integration site; pA—potential polyadenylation signal provided by the SVA 3′ transduction. Chromosome numbers refer to the rhesus genome; a key with the respective numbers in human and orang-utan is provided. MMU, *Macaca mulatta*; HSA, *Homo sapiens*; PPY, *P. (pygmaeus) abelii*. (*B*) Schematic representation of additional lcr16a copies present on orang-utan chromosomes 16 and 11 (ppy_33). Note that in all cases, the 3′ breakpoint localizes to either the SVA or its 3′ transduction. Colored boxes in (*A*) and (*B*) represent lcr16a flanking sequences or flanking SDs (not drawn to scale). Identical colors indicate identical mapping positions (rheMac10) and junctions between the modules and the low copy repeat. The key to the color code is provided in supplementary table S2, Supplementary Material online. For reasons of clarity only immediately flanking modules are shown.

Detailed analysis of the orang-utan BAC sequences revealed that a full-length SVA including the 3′ transduction (composed of MER and L2 sequences; source loci chr17:11,873,831–11,874,225 and chr8:115,061,229–115,061,470; ponAbe3) is flanked by target site duplications (TSD) indicative of L1-mediated retrotransposition in three of the lcr16a copies (ppy_4–6, fig. 2*A*). The source element of this insertion, that is, an SVA carrying the characteristic 3′ transduction, could not be identified in the orang-utan reference genome (ponAbe3) or available BAC sequences. The insertion site is localized in the *NPIP* last exon tandem repeat region. The encoded amino acid variable number of tandem repeats is species-specific in its composition and hyper-expanded in orang-utan when compared with human NPIP (Cantsilieris et al. 2020). Integration of the SVA/3′ transduction occurred in antisense to *NPIP*. The insertion provides a stop codon limiting the length of the last exon to 1,229 amino acids in ppy_4–6. A potential polyadenylation signal is present in the MER part of the SVA 3′ transduction.

The evolutionary history of lcr16a in orang-utan was reconstructed starting with a comparison to the ancestral lcr16a locus as present on rhesus chromosome 20 (rheMac10; chr20:14,713,088–14,735,664; fig. 2*A*). In addition to the *NPIP* gene, the ancestral locus contains an *UBE3A* processed pseudogene (*UBE3A*pp—also present in baboon). An orang-utan lcr16a copy corresponding to the ancestral locus as found on rhesus chromosome 20 (containing the complete *UBE3A*pp but lacking the SVA, i.e., the preintegration allele with respect to the SVA) could not be identified in the available BAC sequences or orang-utan reference genome.

*UBE3A*pp sequence is present in eight of the orang-utan copies (ppy_1–8; fig. 2*A*). In ppy_1–3, the lcr16a 3′ breakpoint maps to *UBE3A*pp—the 5′ end of the processed pseudogene has been lost upon duplication. Ppy_4–8 are characterized by the presence of the full-length *UBE3A*pp. They also share the SVA insertion in the *NPIP* 3′ terminal exon. In addition, a massive expansion of the *NPIP* 3′ terminal repeats to more than 7 kb (compared with 2.1 kb in ppy_1–3) is observed. With regard to the resulting proteins’ last exon this translates into between 1,116 and 1,229 amino acids for ppy_4–8 (delimited by the SVA insertion) compared with 365 amino acids in ppy_1–3.

A median-joining network rooted on the rhesus *UBE3A*pp illustrates the phylogenetic relationships of the *UBE3A*pp-containing orang-utan lcr16a copies (fig. 2*A*). The lcr16a copies carrying a full-length SVA insertion fall into two groups: in ppy_4–6 the order of the 5′ flanking genes corresponds to that found upstream of *NPIP* in rhesus; ppy_7/8 possess a 5′ flanking SD consisting of genes localized downstream of *NPIP* in rhesus (*RRN3*, *NTAN1*, *PDXDC1*–supplementary table S1, Supplementary Material online). Additional copies mapping to chromosome 16 and chromosome 11 (ppy_33) can be subdivided into three groups: ppy_9/10 are characterized by the SVA being truncated in the central VNTR, in ppy_11–16 only the 3′-most 39 bp of the SVA have been retained and in ppy_32 and ppy_33 the 3′ part of the 3′ transduction is the only remnant of the original retrotransposon insertion (fig. 2*B*).

### Interchromosomal Duplicative Transposition to and Amplification on Chromosome 13

**Fig. 3.**
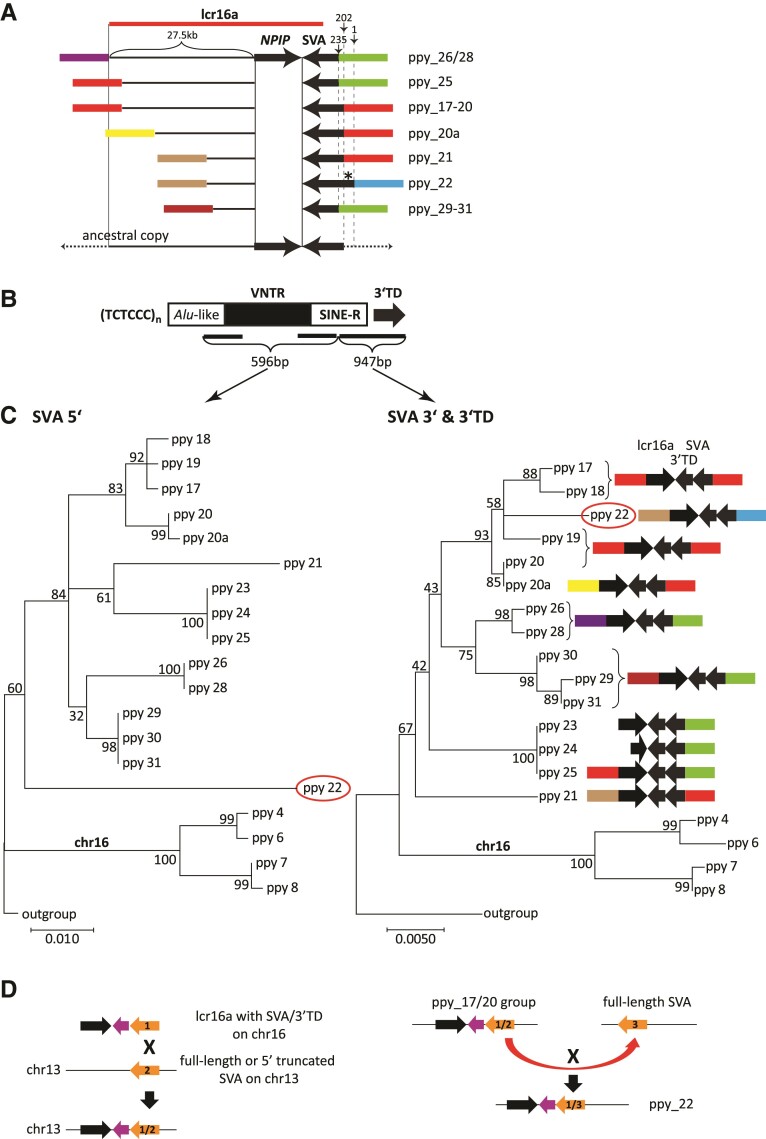
SVA recombination contributes to lcr16a interchromosomal and intrachromosomal mobility in orang-utan. (*A*) Structure and extension of the lcr16a core duplicon in duplication blocks on chromosome 13. Only immediately adjacent flanking sequences/SDs (colored boxes) are shown. Thin horizontal lines denote lcr16a sequence upstream of the *NPIP* coding region (drawn to scale). Numbers above vertical arrows refer to the SVA 5′ end relative to the SVA_A RepeatMasker consensus sequence. Ppy_22 carries a full-length SVA (asterisk) likely resulting from SVA inter-element recombination. Minimal 5′ and 3′ extension of the hypothetical chromosome 13 ancestral lcr16a copy is shown at the bottom. Note that the red boxes at the 5′ and 3′ ends, respectively, represent modules derived from the same locus (separated by 1.1 kb). (*B*) Schematic representation of an SVA element. TCTCCC hexameric repeats at the 5′ end are followed by an *Alu*-like domain, a VNTR and a retrovirus-derived SINE-R. The element present in orang-utan lcr16a carries a 3′ TD. Black bars indicate the regions used in the phylogenetic analysis shown in *C*. Numbers denote the length of the sequences used for the analysis. (*C*) Different phylogenetic relationships of SVA 5′ and 3′ ends suggest recombination as the mechanism of lcr16a interchromosomal (chromosome 16 to chromosome 13) and intrachromosomal (ppy_22 on chromosome 13) duplicative transposition. Maximum likelihood phylogenetic trees (Kimura two-parameter; *n* = 10,000 bootstrap replicates) were generated for SVA sequences up- and downstream of the potential recombination region (for details see main text and supplementary fig. S1, Supplementary Material online). The SVA 5′ region comprises the part of the *Alu*-like domain present in all copies, the first and the last three VNTR subunits as well as the 5′ part of the SINE-R. The central part of the VNTR could not be aligned. The downstream part spans the SINE-R 3′ and the 3′ transduction. Low bootstrap values of, in particular, the interior nodes can be attributed to the relatively short length of the sequences used in the analysis. (*D*) Schematic representation of SVA recombination in interchromosomal (left panel) and intrachromosomal (right panel) lcr16a duplicative transposition. SVAs are shown in orange; the 3′ transduction in purple. The black arrow denotes lcr16a. Numbers indicate the different SVA elements involved in recombination. X denotes recombination events. Colored boxes in (*A*) and (*C*) represent lcr16a flanking sequences or flanking SDs (not drawn to scale). Identical colors indicate identical mapping positions and junctions between the modules and lcr16a at its 5′ and 3′ end, respectively. Modules derived from the same locus are presented in the same color. The key to the color code is provided in supplementary table S3, Supplementary Material online. For reasons of clarity only immediately flanking modules are shown.

Orang-utan is the only great ape in which a considerable number of lcr16a copies is found on a different chromosome than chromosome 16. On chromosome 13, seven copies/groups of copies (ppy_17–ppy_31) can be discerned. In all of them the lcr16a 3′ breakpoint/junction maps to the SVA. At the 5′ end five different breakpoints were used (fig. 3*A*). From the combination of breakpoints in the extant copies (e.g., longest 5′ extension combined with shortest SVA sequence at the 3′ in ppy26/28; fig. 3*A*) it can be concluded that none of them represents the structure of the ancestral lcr16a insertion on chromosome 13.

To investigate whether recombination involving the orang-utan lcr16a SVA has been responsible for translocation of the repeat to chromosome 13, a multiple alignment including the SVAs and 3′ transductions of chromosome 16 and chromosome 13 copies was generated (supplementary fig. S1, Supplementary Material online). A combination of the consensus sequence of ten closely related SVAs, representing the parental subgroup of the lcr16a SVA (supplementary table S4, Supplementary Material online) and of the source sequences of the 3′ transduction, was used as outgroup for comparison. Visual inspection of the alignment revealed that up to the 3′ part of the SINE-R there are no substitutions shared between chromosome 16 and chromosome 13 copies. Thus, the SVA 5′ parts of the chromosome 16 and chromosome 13 copies are unlikely to be derived from the same source. Further downstream a number of substitutions are shared by most/all of the copies—suggesting a common ancestor for this part of the sequence. Separate maximum likelihood phylogenetic trees were constructed for the 5′ and 3′ parts of the SVAs, respectively (fig. 3*B*). The different topologies (fig. 3*C*) reflect the findings from the analysis of the alignment: the 5′ parts of the lcr16a SVAs on chromosomes 16 and 13 do not appear to have a common ancestor, whereas the 3′ parts likely are derived from a common precursor. Theoretically the common ancestry observed for the 3′ part could also be the result of gene conversion replacing an lcr16a fragment in a chromosome 13 copy by the homologous stretch of a chromosome 16 copy. To test this hypothesis, maximum likelihood phylogenetic trees were constructed for two downstream segments: a 304 bp sequence covering the 5′ end of the *NPIP* last exon and *NPIP* exon 2 (not subject to selection in orang-utan (Cantsilieris et al. 2020)). If gene conversion had occurred, then—depending on the length of the converted tract—both or only the tree representing exon 2 should show a topology similar to that seen for the SVA 5′ end. This is not the case (supplementary fig. S2, Supplementary Material online). Thus, provided the gene conversion tract does not extend further than *NPIP* exon 2, gene conversion cannot account for the change in tree topology observed when the SVA 5′ and 3′ are compared. Due to the lack of shared SVA-flanking sequence, a similar analysis cannot be performed for the 5′ end. Whether gene conversion has occurred at the SVA 5′ end can, thus, not be established.

Based on the sequences analyzed, a contribution of SVA–SVA recombination to lcr16a interchromosomal mobility appears possible (fig. 3*D*, left panel). However, as the ancestral copy on chromosome 13 is not available for analysis and only a limited range of sequences could be used for phylogenetic inference, a definitive conclusion cannot be drawn.

Further amplification of lcr16a on chromosome 13 involved two different breakpoints in the SVA *Alu*-like domain and co-mobilization of flanking SDs at the 5′ and/or 3′ end of the lcr16a core (fig. 3*A* and *C*; supplementary table S3, Supplementary Material online). One of the copies, however, does not follow this pattern: ppy_22 resulted from recombination between one of the members of the ppy_17–20 group and a different, full-length SVA (insertion site mapping to hg38 chr13:25,022,161). Again recombination occurred in the 3′ part of the SINE-R region (fig. 3C and D—right panel; supplementary fig. S1, Supplementary Material online). Taken together, SVA–SVA recombination likely contributed to both interchromosomal and intrachromosomal mobility of lcr16a in orang-utan.

### SVA Recombination-mediated Duplicative Transposition of lcr16a in Hominines

**Fig. 4.**
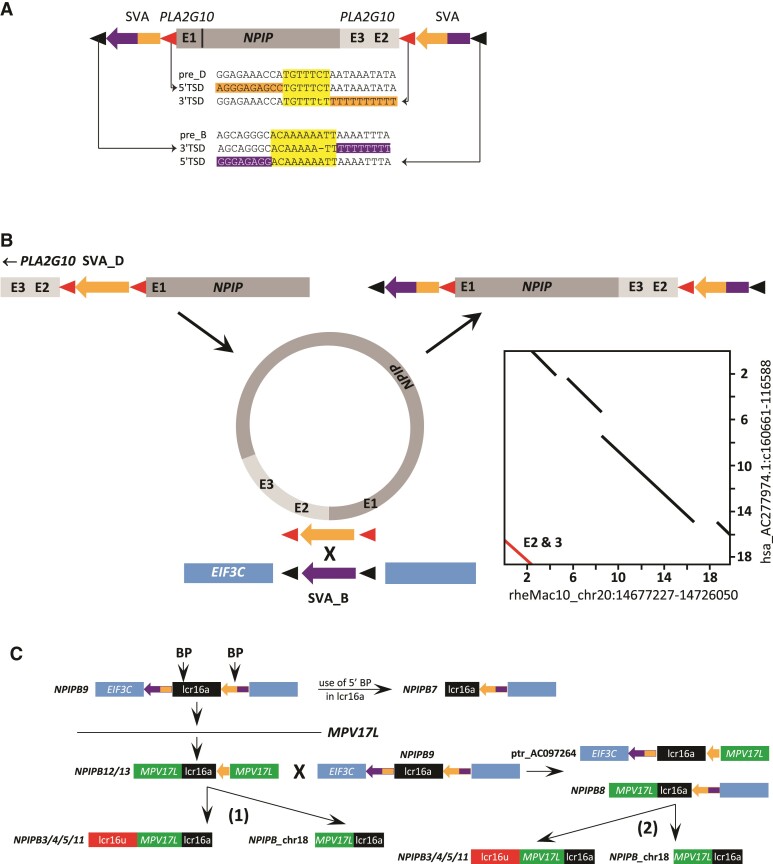
SVA recombination-mediated insertion of an lcr16a circular intermediate is the founding event for the *NPIPB* clade in hominines. (*A*) Schematic representation of lcr16a on AC277974 (hg38 chr16:28,737,136–28,781,237). The core duplicon is flanked by two SVA_B (purple)- SVA_D (orange) chimeric elements, the corresponding TSDs are found at the inner (SVA_D—red triangles) and outer (SVA_B—black triangles) boundaries of the cassette. The SVA preintegration site sequences (pre-B and pre-D) are shown together with the TSDs and surrounding sequences. TSDs are highlighted in yellow, SVA sequences in purple (SVA_B), and orange (SVA_D). Mismatches present in the human TSDs are absent in gorilla and chimpanzee. *PLA2G10* exons (*E*) 3 and 2 are found at the *NPIP* 3′ end. (*B*) SVA recombination-mediated insertion of a circular intermediate can explain the structure presented in (*A*). An lcr16a copy carrying an SVA_D insertion in *PLA2G10* intron 1 circularizes, connecting *PLA2G10* exon 3 and the *NPIP* 3′ end. Reinsertion occurs through recombination between the SVA_D and an SVA_B downstream of *EIF3C*. The resulting copy is characterized by flanking SVA_D/B and SVA_B/D chimeric elements and translocation of *PLA2G10* exons 3 and 2 to the *NPIP* 3′ end. TSDs of the SVA elements are shown as red and black triangles, respectively. The inset shows the dot plot for the alignment of the human copy (AC277974) to the rhesus ancestral locus. The segment corresponding to *PLA2G10* exons 2 and 3 is highlighted in red. E—exon (*C*) Expansion of the *NPIPB* clade of hominine lcr16a. Duplicative transposition of lcr16a with breakpoints (BP) in the lcr16a 5′ and in the 3′ flanking SVA_B/D chimera into the *MPV17L* locus gives rise to the ancestor of *NPIPB12*/*13*. SD structures observed in chimpanzee (ptr) AC097264.4 and human *NPIPB8* can be explained by recombination between the *NPIPB12/13* duplicon and the ancestral copy (with a structure as present in *NPIPB9*). *NPIPB* copies associated with lcr16u and the chromosome 18 copy could be derived from either *NPIPB12/13* (1) or from *NPIPB8* (2). *NPIPB7* could be derived from *NPIPB9* by use of breakpoints in lcr16a and in the 3′ flanking SD. Only flanking modules relevant to the model are shown. Identically colored boxes are derived from the same locus. Where genes are annotated in the reference genome, gene symbols have been included. For detailed mapping data, see supplementary table S5, Supplementary Material online. lcr16u—chromosome 16 low copy repeat u; contains the *SMG1* gene and is frequently found associated with lcr16a (Eichler et al. 2001).

To establish whether there is an association of SVAs with lcr16a in the human genome, SVAs were intersected with SDs on human chromosome 16. Two elements were found overlapping the 3′ ends of lcr16a copies; both in reverse orientation relative to the *NPIP* gene. They display identical 3′ flanking sequences, indicating that they occupy the same position in lcr16a. One of the elements was found to be full-length, the other one is 5′ truncated. Subsequently, BAC sequences harboring the two SVAs were retrieved and analyzed. In two of the clones (AC277974 and AC145285; supplementary table S5, Supplementary Material online) the full-length SVA at the lcr16a 3′ end was found in combination with another full-length element at the lcr16a 5′ end (fig. 4*A*). Analysis of the subfamily affiliation of the two SVAs revealed that they are chimeras combining SVA_B and SVA_D subfamily sequences in a reciprocal manner: the 5′ element (upstream) is SVA_D at its 5′ end and SVA_B at the 3′ end; the 3′ (downstream) SVA displays an SVA_B *Alu*-like region and SVA_D SINE-R (fig. 4*A*; supplementary fig. S3, Supplementary Material online). The outer junctions of the cassette as depicted in figure 4*A* map to a position downstream of *EIF3C*; the inner (lcr16a—SVA) junctions to a position in *PLA2G10* intron 1. TSDs of the *EIF3C* insertion site (black triangles) are found associated with SVA_B sequence (purple) at the 3′ end of the upstream and the 5′ end of the downstream SVA. TSDs matching the *PLA2G10* insertion site (red triangles) are associated with SVA_D sequence (orange) at the 5′ end of the upstream and at the 3′ end of the downstream chimeric element. In addition, *PLA2G10* sequences ranging from exon 3 to intron 1 are found to be shifted to a position downstream of *NPIP* (fig. 4*A*).

Taken together, the structure observed is consistent with a duplication mechanism involving circular intermediates as recently established by Chicote et al. (2020). In the case described here recombination between two SVA elements mediates reintegration of the circular intermediate into the genome (fig. 4*B*): duplication starts with circularization of an lcr16a copy containing an SVA_D insertion in *PLA2G10* intron 1. In the resulting circle, the *NPIP* 3′ end is joined to *PLA2G10* intron 3. At the insertion site, the SVA_D recombined with an SVA_B element facilitating integration of the SVA_D-flanked lcr16a sequence. The resulting structure is the one observed on the BAC clones and is also represented in the human reference genome (hg38 chr16:28,737,136–28,781,237).

Duplication must have occurred in the common hominine ancestor, as a similarly structured lcr16a copy is also found in chimpanzee (AC275224.1) and the two flanking SVAs could be identified in gorilla PacBio reads (supplementary table S5, Supplementary Material online). In the gorilla reference genome (gorGor6) they map at a distance of ∼0.4 Mb (as opposed to 50 kb in the human genome), suggesting that either the original copy has not been retained in gorilla and the two SVAs are now found on separate descendants or that the SD harboring the original copy has not been correctly included into the reference build.

Insertion of the SVA-flanked lcr16a is the seeding event for an entire group of copies (fig. 4*C*; supplementary table S5, Supplementary Material online). In addition to *NPIPB9*, which contains the complete cassette, there are copies in which either the 5′ (chimpanzee AC097264) or the 3′ end (*NPIPB7/8*) of it is preserved. In some copies, a 3′ breakpoint in the SVA is used (the 5′ truncated SVA element identified in the initial screen). It is always found fused to a segment derived from the *MPV17L* locus. The structure observed in *NPIPB12/13* suggests initial integration into *MPV17L* using a 5′ breakpoint upstream of *NPIP*. Subsequent recombination events then gave rise to the “chimeric” copies combining *MPV17L* and *EIF3C* modules. The 5′ *MPV17L* segment is also found in *NPIPB3/4/5/11*, where it has served as accretion point for lcr16u (Eichler et al. 2001) and in the lcr16a localized on chromosome 18.

Taken together, the majority of the *NPIPB* copies in humans (*NPIPB3*-*NPIPB13* and *NPIPB*_chr18) can be traced back to the SVA recombination-mediated duplication event described above (fig. 4*B* and *C*; supplementary table S5, Supplementary Material online). In *NPIPB2* and *NPIPB15*, breakpoints internal to the lcr16a as present in *NPIPB12/13* have been used; flanking SDs derived from the *EIF3C* or *MPV17L* loci are missing in these copies. It is, therefore, not possible to unambiguously establish their descent from one or the other of the *NPIPB* copies.

### Large Direct Repeats Flank SDs in the Human Genome

**Fig. 5.**
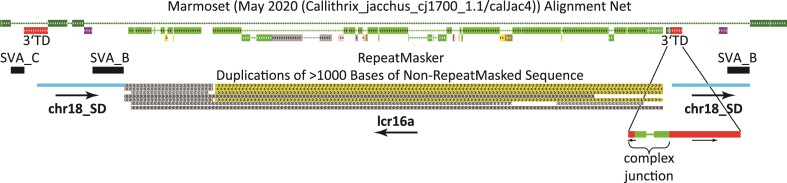
The chromosome 18 lcr16a duplication is flanked by 4 kb direct repeats. A modified UCSC genome browser representation of the lcr16a integration on chromosome 18 is shown. The core duplicon is flanked by ∼4 kb direct repeats (light blue bars; chr18_SD). Breakpoints of the flanking repeats are localized in the 3′ transduction (3′ TD—red bars) of an SVA_C element and directly downstream of an SVA_B element. The inset shows the complex junction at the 3′ lcr16a integration site involving, among others, the insertion of an unrelated DNA segment and the inversion of part of the SVA_C 3′ transduction. The marmoset alignment net is provided to illustrate the different origins of the sequences. The RepeatMasker track has been reduced to show SVA only.

The lcr16a copy on chromosome 18 presents a unique structure: it is flanked by ∼4 kb of directly repeated chromosome 18 sequence (fig. 5; light blue bars—chr18_SD). The duplicated segments are delimited by an SVA_B and the 3′ transduction (3′ TD—shown in red) of an SVA_C element, respectively. In this case, however, the SVAs did not play a role in the integration process of the core duplicon. A subsequent scan of the human genome identified four more instances of SDs flanked by large direct repeats (supplementary table S6, Supplementary Material online): *RhD* (1p36.11) flanked by 9 kb direct repeats and *DPY19L2* (12q14.2) flanked by a duplication of 27 kb have already been described in the literature (Wagner and Flegel 2002; Elinati et al. 2012). The other two instances comprise three genes each—*MMP23A/CDK11A/SLC35E2A* on chromosome 1p36.33 and *CASTOR3/SPDYE/PMS2P1* on chromosome 7q22.1. Based on the UCSC genome browser alignment net and available data from the literature *DPY19L2* has integrated on chromosome 12 prior to mammalian divergence, the *CDK11*-containing duplication dates back to the great ape common ancestor, whereas the other instances observed are hominine-specific (Matassi et al. 1999; Carson et al. 2006; Johnson et al. 2006; Cantsilieris et al. 2020). *DPY19L2* and *NPIPB* represent interchromosomal SDs; the remaining duplications originate from loci of the same chromosome.

Analysis of the junctions between flanking direct repeats and inserted SDs revealed 1–4nt microhomologies and the insertion of extra nucleotides or unrelated segments of DNA (supplementary table S6, Supplementary Material online). The lcr16a copy shows a complex junction at its 3′ end (fig. 5). Insertion-flanking direct repeats could, theoretically, arise when double-strand breaks generate overhanging ends which are then complemented after insertion has occurred—in a manner similar to that suggested for repeats observed at the ends of pericentric inversions (Kehrer-Sawatzki et al. 2005). However, especially the complex junction found at one end of the lcr16a insertion (comprising four different DNA fragments—not shown) cannot be explained by such a mechanism. Only replication-based models involving multiple template switches (FoSTeS/MMBIR—Lee et al. 2007; Hastings et al. 2009) can account for complex junction structures. The microhomologies and insertions of sequence derived from other regions of the genome at the junctions are consistent with these mechanisms.

## Discussion

Early on in the analysis of human SDs an enrichment of *Alu* elements at their boundaries has been observed and a role for *Alu*–*Alu*-mediated recombination in the generation and expansion of SDs has been proposed (Bailey et al. 2003). A recent analysis of lcr16a across primates found *Alu* repeats significantly enriched in both donor and acceptor regions—but not SVAs (Cantsilieris et al. 2020).

Based on the literature, SVA–SVA recombination appears to be a rare event. Re-analysis of the data provided by Lee et al. (2012) revealed that the one event attributed to recombination between SVA elements more likely represents the loss of an SVA element in the human and gorilla lineages. The only other report published postulated SVA–SVA recombination as the cause of a deletion in the *WDR66* gene (Kherraf et al. 2018). Indeed, a global assessment of structural variation in the human genome subsequently identified an SVA insertion at the 3′ end point of the deletion (gnomAD:INS_12_86171; nsv4574453, Collins et al. 2020). A comprehensive analysis of SVA inter-element recombination is not available. Considering the relatively low copy number of SVAs (2,800 in the human genome, Wang et al. 2005) compared with more than 1 million *Alu* elements (Lander et al. 2001), the probability for such events to occur is, however, rather low.

Thus, SVA appeared to be an unlikely candidate to investigate in the context of SD formation and expansion. SVA elements and their gibbon counterparts, LAVA, have previously been shown to coamplify with SDs (RHOT1 transduction group, Damert et al. 2009; Hancks et al. 2009; LAVA_C3, Carbone et al. 2014). The finding that they can provide SD breakpoints and contribute to SD mobility by inter-element recombination was unexpected. In the orang-utan lcr16a copies analyzed here breakpoints in three of the four SVA structural domains are used: in the *Alu*-like region, the VNTR, and the SINE-R. Thus, there is no obvious breakpoint preference determined by sequence/structure. The second breakpoint in the *Alu*-like domain (pos. 235 relative to the RepeatMasker consensus) does, however, coincide with a recombination hotspot described for *Alu* elements (Rüdiger et al. 1995). In case of the first breakpoint in the *Alu*-like region (pos. 202 relative to the RepeatMasker consensus) there are two possible scenarios. Either there has been recombination of the chromosome 16 full-length SVA with a truncated SVA on chromosome 13 (fig. 3*D*—left panel) or recombination occurred between two full-length elements with a subsequent break in the *Alu*-like region upon generation of one of the chromosome 13 copies. Overall, the analysis of lcr16a alone already identified two and one SVA inter-element recombination events in orang-utan and humans, respectively. This suggests that SVA–SVA recombination might be more frequent than expected from previous reports in the literature.

To accurately appreciate SVAs’ contribution to lcr16a mobility, it has to be taken into account that for the extant complex lcr16a duplication blocks the interaction at the insertion site occurred with lcr16a-associated SDs and does not involve SVAs. In orang-utan evolutionary history, however, SVA contributed to lcr16a dispersal and acquisition of core duplicon-associated SDs.

Starting from a few anecdotal observations (Fujimura et al. 2011; Durkin et al. 2012) integration of extrachromosomal circular DNA (eccDNA) has only been recognized as a mechanism of SD formation in the last years. Recently, SDs were found to be significantly overrepresented in sperm eccDNA (Mouakkad-Montoya et al. 2021). To date, only a relatively small number of duplication events generated by this mechanism has been characterized (Chicote et al. 2020; Takahashi and Innan 2020). Fundamental questions regarding circularization and integration are still unanswered. Based on the analysis published homology-based mechanisms did not appear to play a role in the integration process (Chicote et al. 2020). The structure of the *NPIPB9* lcr16a copy analyzed here (fig. 4*A*), however, is consistent with the reintegration of a circular intermediate through recombination between homeologous SVA elements. Whether this has been a unique event remains to be seen. Based on the analysis of descendent copies (see fig. 4*C*, supplementary table S5, Supplementary Material online) it is, however, very likely that this particular event stands at the base of the entire lcr16a/*NPIPB* expansion in African apes. Much of the positive selection detected in gorilla, human, and chimpanzee can be attributed to the *NPIPB* subfamily (Cantsilieris et al. 2020). It is tempting to speculate that SVA-mediated transposition to a more distal location, by increasing the distance to other lcr16a (*NPIPA*) copies, decreased the probability of homogenizing gene conversion and, thus, permitted independent evolution of *NPIPB*. Taken together, the data presented here reveal a previously unrecognized role for the hominid-specific SVA retrotransposon in the mobilization of the lcr16a core duplicon.

Up to now, there had only been two isolated observations of gene duplicates flanked by large direct repeats (*RHD* and *DPY19L2*, Wagner and Flegel 2002; Elinati et al. 2012). The identification of three more instances in the study presented here (*NPIPBP*_chr18, *MMP23A/CDK11A/SLC35E2A*, and *CASTOR3/SPDYE/PMS2P1*) raises the question of whether this might represent a more general phenomenon. In the reference genome additional events of this type may be hidden by complex rearrangements at sites of SD, especially in the subtelomeric and pericentromeric regions—and, therefore, difficult to detect. In addition, SDs flanked by direct repeats are prone to deletion involving NAHR (nonallelic homologous recombination) between the flanking sequences, leaving behind a single chimeric copy of the original flanking direct repeats. Indeed, such deletion events have been described for all five cases reported here—either as duplication-specific observations (Wagner and Flegel 2000; Elinati et al. 2012) or in the framework of whole-genome structural variation surveys (Korbel et al. 2007; The International HapMap 3 Consortium 2010; Mills et al. 2011; Wong et al. 2013, dgv accession numbers are provided in supplementary table S6, Supplementary Material online). Interestingly, a recent comprehensive survey of structural variants (SVs) in the human genome does not list the combination of insertion and duplication observed here among the subclasses of complex SVs (Collins et al. 2020); neither are there, to my knowledge, any reports of disease-associated SVs of this type. Thus, de novo generation of such duplication/insertion structures appears to be extremely rare or it escapes detection due to the simultaneous presence of duplication and insertion signatures at the same site.

## Materials and Methods

### BACs, Mapping, and Identification of Repeat-flanked SDs

BACs analyzed are those reported in Cantsilieris et al. (2020). Additional BACs spanning lcr16a copies were identified by BLAST using the *NPIPA* coding region or SVA sequences as queries in searches against the NCBI nucleotide (nr/nt) and htgs databases. Sequences were mapped against the rhesus (rheMac10), human (hg38), and orang-utan (ponAbe3, Susie_PAB_pri, Vollger et al. 2022) genomes. All mapping was done using BLAT at the UCSC genome browser. Susie_PAB_pri sequences were analyzed using the NCBI Genome Workbench (Kuznetsov and Bollin 2021). Where possible and appropriate, conclusions based on the analysis of BAC sequences were cross-checked against the respective reference genomes and/or available sequence read archives (PacBio) at NCBI. The dot plot was generated using YASS (Noe and Kucherov 2005) with repeat-masked input sequences. SDs flanked by large direct repeats were identified by visual screening using the UCSC genome browser SD track and nonoverlapping 0.5 Mb windows.

### Phylogenetic and Recombination Analysis

All multiple alignments were calculated using ClustalW (Thompson et al. 1994) in Bioedit. The median-joining network was constructed using Network (Bandelt et al. 1999) with default parameters (equal weight for all positions, no preprocessing, or postprocessing) and external rooting. Maximum likelihood phylogenetic trees were generated with MEGA X (Kumar et al. 2018) (Kimura two-parameter; *n* = 10,000 bootstrap replicates). For analysis of SVA recombination in orang-utan, the SVAs were separated at the point of an internal 34/67 bp sequence duplication in ppy_17–20 which divides the potential recombination interval (between last chromosome 16-specific substitution and first chromosome 16—chromosome 13-shared substitution—306 bp) approximately in half.

## Supplementary Material

Supplementary data are available at *Molecular Biology and Evolution* online.

## Supplementary Material

msac103_Supplementary_Data

## Data Availability

No new data were generated for this research. The data underlying this article are available in public repositories and were processed as described in Materials and Methods.

## References

[msac103-B2] Bailey JA, Liu G, Eichler EE. 2003. An Alu transposition model for the origin and expansion of human segmental duplications. Am J Hum Genet. 73:823–834. 14505274 10.1086/378594 PMC1180605

[msac103-B3] Bailey JA, Yavor AM, Massa HF, Trask BJ, Eichler EE. 2001. Segmental duplications: organization and impact within the current human genome project assembly. Genome Res. 11:1005–1017. 11381028 10.1101/gr.187101 PMC311093

[msac103-B4] Bandelt HJ, Forster P, Röhl A. 1999. Median-joining networks for inferring intraspecific phylogenies. Mol Biol Evol. 16:37–48. 10331250 10.1093/oxfordjournals.molbev.a026036

[msac103-B5] Batzer MA, Deininger PL. 2002. Alu repeats and human genomic diversity. Nat Rev Genet. 3:370–379. 11988762 10.1038/nrg798

[msac103-B6] Bekpen C, Baker C, Hebert MD, Sahin HB, Johnson ME, Celik A, Mullikin JC, Program NCS, Eichler EE. 2017. Functional characterization of the Morpheus gene family. bioRxiv. 10.1101/116087.

[msac103-B7] Boone PM, Yuan B, Campbell IM, Scull JC, Withers MA, Baggett BC, Beck CR, Shaw CJ, Stankiewicz P, Moretti P, et al 2014. The Alu-rich genomic architecture of SPAST predisposes to diverse and functionally distinct disease-associated CNV alleles. Am J Hum Genet. 95:143–161. 25065914 10.1016/j.ajhg.2014.06.014 PMC4129405

[msac103-B8] Cantsilieris S, Sunkin SM, Johnson ME, Anaclerio F, Huddleston J, Baker C, Dougherty ML, Underwood JG, Sulovari A, Hsieh P, et al 2020. An evolutionary driver of interspersed segmental duplications in primates. Genome Biol. 21:202. 32778141 10.1186/s13059-020-02074-4 PMC7419210

[msac103-B9] Carbone L, Harris RA, Gnerre S, Veeramah KR, Lorente-Galdos B, Huddleston J, Meyer TJ, Herrero J, Roos C, Aken B, et al 2014. Gibbon genome and the fast karyotype evolution of small apes. Nature 513:195–201. 25209798 10.1038/nature13679 PMC4249732

[msac103-B10] Carson AR, Cheung J, Scherer SW. 2006. Duplication and relocation of the functional DPY19L2 gene within low copy repeats. BMC Genomics 7:45. 16526957 10.1186/1471-2164-7-45 PMC1475853

[msac103-B11] Chicote JU, López-Sánchez M, Marquès-Bonet T, Callizo J, Pérez-Jurado LA, García-Espana A. 2020. Circular DNA intermediates in the generation of large human segmental duplications. BMC Genomics 21:593. 32847497 10.1186/s12864-020-06998-w PMC7450558

[msac103-B12] Collins RL, Brand H, Karczewski KJ, Zhao X, Alföldi J, Francioli LC, Khera AV, Lowther C, Gauthier LD, Wang H, et al 2020. A structural variation reference for medical and population genetics. Nature 581:444–451. 32461652 10.1038/s41586-020-2287-8 PMC7334194

[msac103-B13] Damert A . 2018. Phylogenomic analysis reveals splicing as a mechanism of parallel evolution of non-canonical SVAs in hominine primates. Mob DNA 9:30. 30237828 10.1186/s13100-018-0135-2 PMC6139936

[msac103-B14] Damert A, Raiz J, Horn AV, Lower J, Wang H, Xing J, Batzer MA, Löwer R, Schumann GG. 2009. 5'-Transducing SVA retrotransposon groups spread efficiently throughout the human genome. Genome Res. 19:1992–2008. 19652014 10.1101/gr.093435.109 PMC2775593

[msac103-B15] Durkin K, Coppieters W, Drögemüller C, Ahariz N, Cambisano N, Druet T, Fasquelle C, Haile A, Horin P, Huang L, et al 2012. Serial translocation by means of circular intermediates underlies colour sidedness in cattle. Nature 482:81–84. 22297974 10.1038/nature10757

[msac103-B16] Eichler EE, Johnson ME, Alkan C, Tuzun E, Sahinalp C, Misceo D, Archidiacono N, Rocchi M. 2001. Divergent origins and concerted expansion of two segmental duplications on chromosome 16. J Hered. 92:462–468. 11948212 10.1093/jhered/92.6.462

[msac103-B17] Elinati E, Kuentz P, Redin C, Jaber S, Vanden Meerschaut F, Makarian J, Koscinski I, Nasr-Esfahani MH, Demirol A, Gurgan T, et al 2012. Globozoospermia is mainly due to DPY19L2 deletion via non-allelic homologous recombination involving two recombination hotspots. Hum Mol Genet. 21:3695–3702. 22653751 10.1093/hmg/dds200

[msac103-B18] Fujimura K, Conte MA, Kocher TD. 2011. Circular DNA intermediate in the duplication of Nile tilapia vasa genes. PLoS One 6:e29477. 22216289 10.1371/journal.pone.0029477 PMC3245284

[msac103-B19] Han K, Lee J, Meyer TJ, Wang J, Sen SK, Srikanta D, Liang P, Batzer MA. 2007. Alu recombination-mediated structural deletions in the chimpanzee genome. PLoS Genet. 3:1939–1949. 17953488 10.1371/journal.pgen.0030184 PMC2041999

[msac103-B20] Hancks DC, Ewing AD, Chen JE, Tokunaga K, Kazazian HH Jr. 2009. Exon-trapping mediated by the human retrotransposon SVA. Genome Res. 19:1983–1991. 19635844 10.1101/gr.093153.109 PMC2775590

[msac103-B21] Hastings PJ, Ira G, Lupski JR. 2009. A microhomology-mediated break-induced replication model for the origin of human copy number variation. PLoS Genet. 5:e1000327. 19180184 10.1371/journal.pgen.1000327 PMC2621351

[msac103-B22] Johnson ME, NISC Comparative Sequencing Program, Cheng Z, Morrison VA, Scherer S, Ventura M, Gibbs RA, Green ED, Eichler EE. 2006. Recurrent duplication-driven transposition of DNA during hominoid evolution. Proc Natl Acad Sci U S A. 103:17626–17631. 17101969 10.1073/pnas.0605426103 PMC1693797

[msac103-B23] Johnson ME, Viggiano L, Bailey JA, Abdul-Rauf M, Goodwin G, Rocchi M, Eichler EE. 2001. Positive selection of a gene family during the emergence of humans and African apes. Nature 413:514–519. 11586358 10.1038/35097067

[msac103-B24] Kehrer-Sawatzki H, Sandig CA, Goidts V, Hameister H. 2005. Breakpoint analysis of the pericentric inversion between chimpanzee chromosome 10 and the homologous chromosome 12 in humans. Cytogenet Genome Res. 108:91–97. 15545720 10.1159/000080806

[msac103-B25] Kherraf ZE, Amiri-Yekta A, Dacheux D, Karaouzène T, Coutton C, Christou-Kent M, Martinez G, Landrein N, Le Tanno P, Fourati Ben Mustapha S, et al 2018. A homozygous ancestral SVA-insertion-mediated deletion in WDR66 induces multiple morphological abnormalities of the sperm flagellum and male infertility. Am J Hum Genet. 103:400–412. 30122540 10.1016/j.ajhg.2018.07.014 PMC6128304

[msac103-B26] Korbel JO, Urban AE, Affourtit JP, Godwin B, Grubert F, Simons JF, Kim PM, Palejev D, Carriero NJ, Du L, et al 2007. Paired-end mapping reveals extensive structural variation in the human genome. Science 318:420–426. 17901297 10.1126/science.1149504 PMC2674581

[msac103-B27] Kumar S, Stecher G, Li M, Knyaz C, Tamura K. 2018. MEGA X: molecular evolutionary genetics analysis across computing platforms. Mol Biol Evol. 35:1547–1549. 29722887 10.1093/molbev/msy096 PMC5967553

[msac103-B28] Kuznetsov A, Bollin CJ. 2021. NCBI genome workbench: desktop software for comparative genomics, visualization, and GenBank data submission. Methods Mol Biol. 2231:261–295. 33289898 10.1007/978-1-0716-1036-7_16

[msac103-B29] Lander ES, Linton LM, Birren B, Nusbaum C, Zody MC, Baldwin J, Devon K, Dewar K, Doyle M, FitzHugh W, et al 2001. Initial sequencing and analysis of the human genome. Nature 409:860–921. 11237011 10.1038/35057062

[msac103-B30] Lee JA, Carvalho CM, Lupski JR. 2007. A DNA replication mechanism for generating nonrecurrent rearrangements associated with genomic disorders. Cell 131:1235–1247. 18160035 10.1016/j.cell.2007.11.037

[msac103-B31] Lee J, Ha J, Son S-Y, Han K. 2012. Human genomic deletions generated by SVA-associated events. Comp Funct Genomics 2012:807270. 22666087 10.1155/2012/807270 PMC3362811

[msac103-B32] Loftus BJ, Kim U-J, Sneddon VP, Kalush F, Brandon R, Fuhrmann J, Mason T, Crosby ML, Barnstead M, Cronin L, et al 1999. Genome duplications and other features in 12 Mb of DNA sequence from human chromosome 16p and 16q. Genomics 60:295–308. 10493829 10.1006/geno.1999.5927

[msac103-B33] Lupan I, Bulzu P, Popescu O, Damert A. 2015. Lineage specific evolution of the VNTR composite retrotransposon central domain and its role in retrotransposition of gibbon LAVA elements. BMC Genomics 16:389. 25981446 10.1186/s12864-015-1543-z PMC4432496

[msac103-B34] Marques-Bonet T, Eichler EE. 2009. The evolution of human segmental duplications and the core duplicon hypothesis. Cold Spring Harb Symp Quant Biol. 74:355–362. 19717539 10.1101/sqb.2009.74.011 PMC4114149

[msac103-B35] Matassi G, Chérif-Zahar B, Pesole G, Raynal V, Cartron J-P. 1999. The members of the RH gene family (RH50 and RH30) followed different evolutionary pathways. J Mol Evol. 48:151–159. 9929383 10.1007/pl00006453

[msac103-B36] Mills RE, Walter K, Stewart C, Handsaker RE, Chen K, Alkan C, Abyzov A, Yoon SC, Ye K, Cheetham RK, et al 2011. Mapping copy number variation by population-scale genome sequencing. Nature 470:59–65. 21293372 10.1038/nature09708 PMC3077050

[msac103-B37] Mouakkad-Montoya L, Murata MM, Sulovari A, Suzuki R, Osia B, Malkova A, Katsumata M, Giuliano AE, Eichler EE, Tanaka H. 2021. Quantitative assessment reveals the dominance of duplicated sequences in germline-derived extrachromosomal circular DNA. Proc Natl Acad Sci U S A. 118:e2102842118. 34789574 10.1073/pnas.2102842118 PMC8617514

[msac103-B38] Noe L, Kucherov G. 2005. YASS: enhancing the sensitivity of DNA similarity search. Nucleic Acids Res. 33:W540–W543. 15980530 10.1093/nar/gki478 PMC1160238

[msac103-B39] Rüdiger NS, Gregersen N, Kielland-Brandt MC. 1995. One short well conserved region of *Alu*-sequences is involved in human gene rearrangements and has homology with prokaryotic *chi*. Nucleic Acids Res. 23:256–260. 7862530 10.1093/nar/23.2.256 PMC306663

[msac103-B40] Takahashi KK, Innan H. 2020. Duplication with structural modification through extrachromosomal circular and lariat DNA in the human genome. Sci Rep. 10:7150. 32345992 10.1038/s41598-020-63665-6 PMC7188851

[msac103-B1] The International HapMap 3 Consortium . 2010. Integrating common and rare genetic variation in diverse human populations. Nature 467:52–58. 20811451 10.1038/nature09298 PMC3173859

[msac103-B41] Thompson JD, Higgins DG, Gibson TJ. 1994. CLUSTAL W: improving the sensitivity of progressive multiple sequence alignment through sequence weighting, position-specific gap penalties and weight matrix choice. Nucleic Acids Res. 22:4673–4680. 7984417 10.1093/nar/22.22.4673 PMC308517

[msac103-B42] Vollger MR, Guitart X, Dishuck PC, Mercuri L, Harvey WT, Gershman A, Diekhans M, Sulovari A, Munson KM, Lewis AP, et al 2022. Segmental duplications and their variation in a complete human genome. Science 376:eabj6965. 35357917 10.1126/science.abj6965 PMC8979283

[msac103-B43] Wagner FF, Flegel WA. 2000. RHD gene deletion occurred in the Rhesus box. Blood 95:3662–3668. 10845894

[msac103-B44] Wagner FF, Flegel WA. 2002. RHCE represents the ancestral RH position, while RHD is the duplicated gene. Blood 99:2272–2273. 11902138 10.1182/blood-2001-12-0153

[msac103-B45] Wang H, Xing J, Grover D, Hedges DJ, Han K, Walker JA, Batzer MA. 2005. SVA elements: a hominid-specific retroposon family. J Mol Biol. 354:994–1007. 16288912 10.1016/j.jmb.2005.09.085

[msac103-B46] Wong L-P, Ong RT-H, Poh W-T, Liu X, Chen P, Li R, Lam KK-Y, Pillai NE, Sim K-S, Xu H, et al 2013. Deep whole-genome sequencing of 100 southeast Asian Malays. Am J Hum Genet. 92:52–66. 23290073 10.1016/j.ajhg.2012.12.005 PMC3542459

[msac103-B47] Xing J, Wang H, Belancio VP, Cordaux R, Deininger PL, Batzer MA. 2006. Emergence of primate genes by retrotransposon-mediated sequence transduction. Proc Natl Acad Sci U S A. 103:17608–17613. 17101974 10.1073/pnas.0603224103 PMC1693794

