# Supplementary material for: SVA Retrotransposons and a Low Copy Repeat in Humans and Great Apes: A Mobile Connection
Source: Mol Biol Evol. 2022 May 16;39(5):msac103. doi: 10.1093/molbev/msac103 (PMC9132208; doi:10.1093/molbev/msac103)

Supplementary figures

Supplementary figure S1 **Multiple alignment of orangutan lcr16a-associated SVAs and 3’ transduced sequences.**

Supplementary figure S2 **Maximum likelihood phylogenetic trees of *NPIP* exon sequences.**

Supplementary figure S3 **Multiple alignment of the chimeric SVAs (5’ D/B and 3’ B/D) resulting from integration of the lcr16a circular intermediate.**

Supplementary figure S1: Multiple alignment of orang-utan lcr16a-associated SVAs and 3' transduced sequences. The alignment includes the first and last three subunits of the central VNTR domain. Groups of chr13 lcr16a copies are colour-coded. For details on the construction of the outgroup consensus see main text.

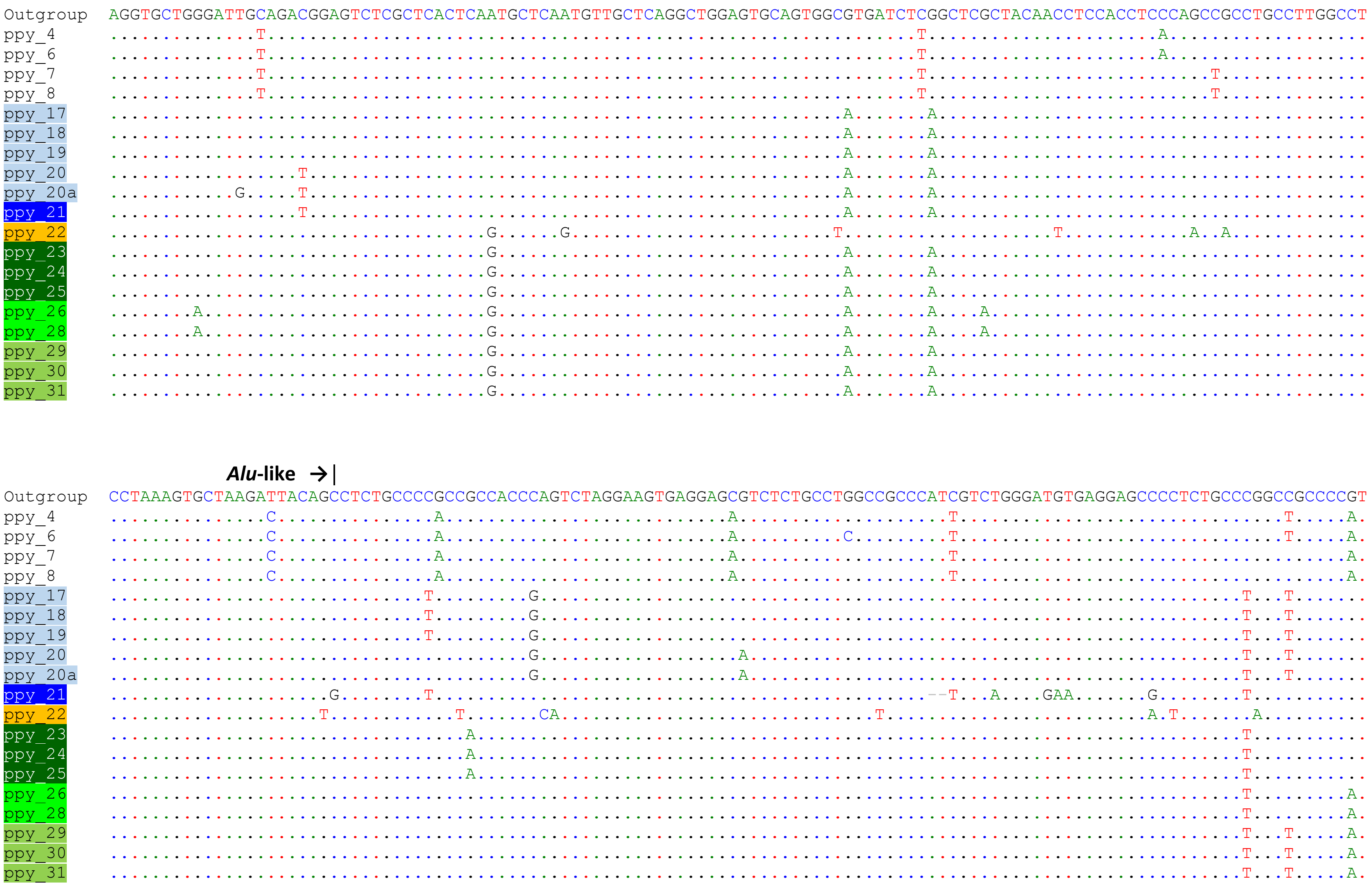

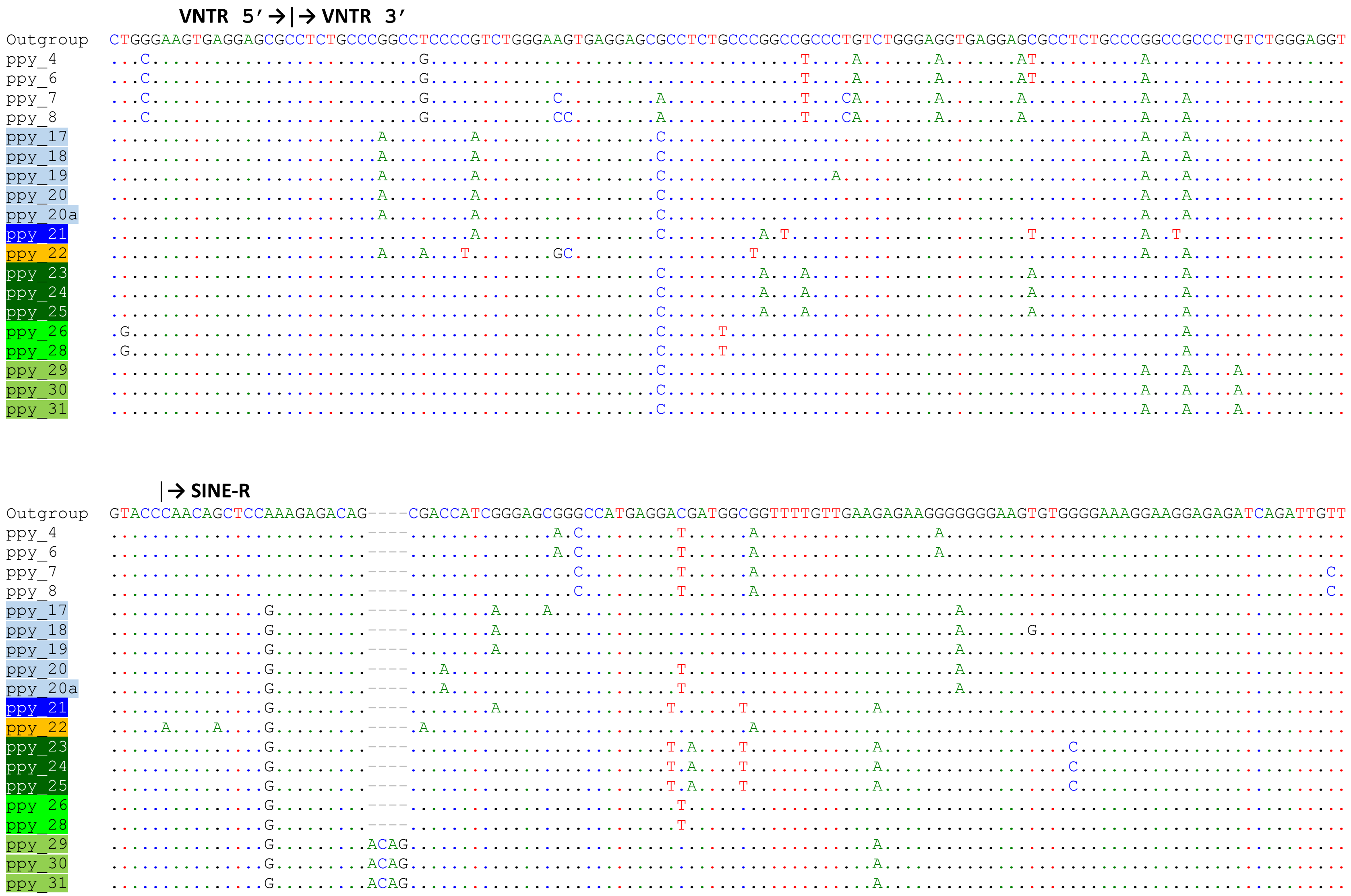
VNTR 5′→|→VNTR 3′
Outgroup CTGGGAAGTGAGGAGCGCCTCTGCCCGGCCTCCCCGTCTGGGAAGTGAGGAGCGCCTCTGCCCGGCCGCCCTGTCTGGGAGGTGAGGAGCGCCTCTGCCCGGCCGCCCTGTCTGGGAGGT
ppy_4
ppy_6
ppy_7
ppy_8
ppy_17
ppy_18
ppy_19
ppy_20
ppy_20a
ppy_21
ppy_22
ppy_23
ppy_24
ppy_25
ppy_26
ppy_28
ppy_29
ppy_30
ppy_31
|→SINE-R
Outgroup GTACCCAACAGCTCCAAAGAGACAG----CGACCATCGGGAGCGGGCCATGAGGACGATGGCGGTTTTGTTGAAGAGAAGGGGGGGAAGTGTGGGGAAAGGAAGGAGAGATCAGATTGTT
ppy_4
ppy_6
ppy_7
ppy_8
ppy_17
ppy_18
ppy_19
ppy_20
ppy_20a
ppy_21
ppy_22
ppy_23
ppy_24
ppy_25
ppy_26
ppy_28
ppy_29
ppy_30
ppy_31

```
Outgroup   GCTGTGTCTGTGTAGAAAGAGGTGGGCATAGGAGACTCCATTTTGTTCTGACTAGGAGAAATTCTTCTGCCTTGGGATGCTGTTGATCTATGGCCTTTCCCCCAGCCCCCTGCTCT----
ppy_4      .........C..........................................................................................................----
ppy_6      ....................................................................................................................----
ppy_7      ....G...............................................................................................................----
ppy_8      ....G...............................................................................................................----
ppy_17     ....................................................................................................................----
ppy_18     ...................................................................................................................C----
ppy_19     ....................................................................................................................----
ppy_20     ....................................................................................................................CTGA
ppy_20a    ....................................................................................................................CTGA
ppy_21     ....................................................................................................................----
ppy_22     .............................................................................................................A......----
ppy_23     ..........C..........................................G..............................................................----
ppy_24     ..........C..........................................G..............................................................----
ppy_25     ..........C..........................................G..............................................................----
ppy_26     .....A...........................................................................................G...........G......----
ppy_28     .....A...........................................................................................G...........G......----
ppy_29     ....................................................................................................................----
ppy_30     ....................................................................................................................----
ppy_31     ....................................................................................................................----

Outgroup   ---------------------------------------------------------------CTGAAACATGTGCTGTGTCAACTCAGGGTTAAATGGATTAAGGGCGGTGCAAGATGT
ppy_4      ---------------------------------------------------------------.................G.......................................
ppy_6      ---------------------------------------------------------------.................G.......................................
ppy_7      ---------------------------------------------------------------.........................................................
ppy_8      ---------------------------------------------------------------.........................................................
ppy_17     -----------------------------ATGATCTATGGCCTTTCCCCCAGCCCCCTGCTCT........---..............................................
ppy_18     -----------------------------ATGATCTATGGCCTTTCCCCCAGCCCCCTGCTCT........---..............................................
ppy_19     -----------------------------ATGATCTATGGCCTTTCCCCCAGCCCCCTGCTCT........---..............................................
ppy_20     AACAGGGGTTTCAGAGAGCCCCCTGCTCTATGATCTATGGCCTTTCCCCCAGCCCCCTGCTCT........---.................................T............
ppy_20a    AACAGGGGTTTCAGAGAGCCCCCTGCTCTATGATCTATGGCCTTTCCCCCAGCCCCCTGCTCT........---.................................T............
ppy_21     ---------------------------------------------------------------.........................................................
ppy_22     ---------------------------------------------------------------.........................................................
ppy_23     ---------------------------------------------------------------.............................................A...........
ppy_24     ---------------------------------------------------------------.............................................A...........
ppy_25     ---------------------------------------------------------------.............................................A...........
ppy_26     ---------------------------------------------------------------.........................................................
ppy_28     ---------------------------------------------------------------.........................................................
ppy_29     ---------------------------------------------------------------.......................G.................................
ppy_30     ---------------------------------------------------------------.......................G.................................
ppy_31     ---------------------------------------------------------------.......................G.................................
```

```
Outgroup  GCTTTGTTAAACAGATGCTTGAAGGCAGCATGCTCTTTAAGAGTCATCACCACTCCCTAATCTCAAGTACCCAGGGGCACAAACACTGCAGAAGGCCGCAGGGACCTCTGCCTAGGAAAA
ppy_4     ............................................................................A...........................................
ppy_6     ............................................................................A...........................................
ppy_7     ............................G...............................................A...................T.......................
ppy_8     ............................G...............................................A...........................................
ppy_17    ............................................................................A...........................................
ppy_18    ............................................................................A...........................................
ppy_19    ............................................................................A...........................................
ppy_20    ............................................................................A...........................................
ppy_20a   ............................................................................A...........................................
ppy_21    ...C........................................................................A....................A......................
ppy_22    ...........................................A................................A...........................................
ppy_23    ................A.......................................................C........................T......................
ppy_24    ................A.......................................................C........................T......................
ppy_25    ................A.......................................................C........................T......................
ppy_26    ............................................................................A........................A..................
ppy_28    ............................................................................A........................A..................
ppy_29    ...C........................................................................A...........................................
ppy_30    ...C........................................................................A...........................................
ppy_31    ...C........................................................................A...........................................
```

|→ Mer

```
Outgroup  CCAGAGACCTTTGTTCATGTGTTTATCTCCTGACCTTCTCTCCACTATTATCCTATGACCCTGCCATATCCCCCTCTCCGAGAAACACCCAAGAATGATCAATAAATACTTAATAAATTT
ppy_4     ......................................C.......G...............C.................................C....-.........C........
ppy_6     ......................................C.......G...............T.................................C....-.........C........
ppy_7     ......................................C.......G.................................................C..............C..T.....
ppy_8     ......................................C.......G.................................................C..............C..T.....
ppy_17    ................................................C.............C...............T..A..............C..............C........
ppy_18    ................................................C.............C..................A..............C..............C........
ppy_19    ................................................C.............C..................A..............C..............C........
ppy_20    ................................................C................................A..............C..............C........
ppy_20a   ................................................C................................A..............C..............C........
ppy_21    ..............................................................C.................................C..............C........
ppy_22    ................................................C.............C...............T..A..............C..............C........
ppy_23    ...........................................................................................G....C..............C........
ppy_24    ...........................................................................................G....C..............C........
ppy_25    ...........................................................................................G....C..............C........
ppy_26    ................................................C.....C...........C............................................C........
ppy_28    ................................................C.....C...........C............................................C........
ppy_29    ................................................C.....C...........C.........................A...C..............C........
ppy_30    ................................................C.....C...........C.............................C..............C........
ppy_31    ................................................C.....C...........C.........................A...C..............C........
```

```
Outgroup  CTGGAATTCACATTTTAAAAGTAAAAAACCAAACAGGTGAATTTAACTTTCATTTAACTCAATATATCTAAAATACCAAATATCATTTCAATATATAATAAATATAAAATTACTAGACTA
ppy_4     ....................A.............................T.....................................................................C
ppy_6     ...........G........A.............................T.....................................................................C
ppy_7     ....................A.........................T...T.....................................................................
ppy_8     ....................A.........................T...T.....................................................................
ppy_17    ..................................................T.................C...................................................
ppy_18    ..................................................T.................C...................................................
ppy_19    ..................................................T.................C...................................................
ppy_20    ..................................................T.................C...................................................
ppy_20a   ..................................................T.................C...................................................
ppy_21    ..................................................T..................................................................T..
ppy_22    ..................................................T.................C...................................................
ppy_23    ..............................C...................T.....................................................................
ppy_24    ..............................C...................T.....................................................................
ppy_25    ..............................C...................T.....................................................................
ppy_26    ..................................................T.....................................................................
ppy_28    .........................................A........T.....................................................................
ppy_29    ..................................................T.....................................................................
ppy_30    ..................................................T.....................................................................
ppy_31    ..................................................T.....................................................................
```

```
Outgroup  TTTTACAGTTTTTGGTCTTATGGCTTTGAAATGTGGTGTGTAATTTAAGCTTATAGCACATCTCAATTTGGACTATCGCATTTCAAGGCTCGGTAGTCACCTGTCATTAATGGCTACTGT
ppy_4     ...........C.................C.......................................A.................T................................
ppy_6     ...........C.................T.................................A.....A.................T................................
ppy_7     .............................C..............................................T...........................................
ppy_8     .............................C..............................................T...........................................
ppy_17    ...............C...............C........................................................................................
ppy_18    ...............C...............C........................................................................................
ppy_19    ...C...........C...............C........................................................................................
ppy_20    ...............C...............C........................................................................................
ppy_20a   ...............C...............C........................................................................................
ppy_21    ...............................C...........................................................AA...........................
ppy_22    ..........C....................C.........................................................C..............................
ppy_23    ...............C............G..C........................................................................................
ppy_24    ...............C............G..C........................................................................................
ppy_25    ...............C............G..C........................................................................................
ppy_26    ...............C...............C........................................................................................
ppy_28    ...............C...............C........................................................................................
ppy_29    ...............................C........................................................................................
ppy_30    ...............................C........................................................................................
ppy_31    ...............................C........................................................................................
```

```
Outgroup  ATTGGGCATTAAAAGTCTAAGAACTTGTCTTCTGTAGTTTTCCTACATAGGGAGATAACTTCTGGGTAGTCTGTTGTCAGTATATGGCCCTTTGAAAATGATGCCTAGTTGTTATTGAAA
ppy_4     .A......................................................T.......C.....................................................
ppy_6     .A......................................................T.......C.....................................................
ppy_7     .A....T..........C......................................T.......C.....................................................
ppy_8     .A....T..........C......................................T.......C.....................................................
ppy_17    ........................................................T.......C...................................................T.
ppy_18    ........................................................T.......C...................................................T.
ppy_19    ................................................G.......T.......C.....................................................
ppy_20    ........................................................T.......C.....................................................
ppy_20a   ........................................................T.......C.....................................................
ppy_21    G.......................................................T.......C.....................................................
ppy_22    ........................................................T.......C.....................................................
ppy_23    ..............A.........................................T.......C.....................................................
ppy_24    ..............A.........................................T.......C.....................................................
ppy_25    ..............A.........................................T.......C.....................................................
ppy_26    ..............A.........................................T....A..C.....................................................
ppy_28    ..............A.........................................T....A..C.....................................................
ppy_29    ..............A.........................................T.......C.............................................---.......
ppy_30    ..............A.........................................T.......C.............................................---.......
ppy_31    ..............A.........................................T.......C.............................................---.......
```

```
                                        |→ L2
Outgroup  CACAATAAATTGGCCATTGGGCATTTGGTACAGAGCTTACTCTATACAGGCATTGGGGGCTAAGCAGTGAAAAAAATCTAAGCACCTACACTCAAGGAGTTTATAGTCAAAGTGACTGAA
ppy_4     ..G.....................................................................................................................
ppy_6     ..G.....................................................................................................................
ppy_7     ..G.....................................................................................................................
ppy_8     ..G.....................................................................................................................
ppy_17    ..G.............................A........................................T..................A...........-...............
ppy_18    ..G.............................A........G...............................T..................A...........-...............
ppy_19    ..G.............................A...........................................................A...........................
ppy_20    ..G.............................A...........................................................A...........................
ppy_20a   ..G.............................A...........................................................A...........................
ppy_21    ..G................................A....................................................................................
ppy_22    ..G.............................A...........................................................A..........C................
ppy_23    ..G.....................................................................................................................
ppy_24    ..G.....................................................................................................................
ppy_25    ..G.....................................................................................................................
ppy_26    ..G.....................................................................................................................
ppy_28    ..G.....................................................................................................................
ppy_29    ..G....................................................A...........G......................A...........................
ppy_30    ..G....................................................A................................................................
ppy_31    ..G....................................................A...........G....................................................
```

```
Outgroup  ATACAAATAAACTAGCAGTTCTAACAGTGAGAAAAGCTTAGGGTTCTGGGAGTGGGGGAGTGTTGAAAAAATGCGTATCATATGATAGGTGCACAATCAATACTTAAATGAATTTGTTTG
ppy_4     ........................A.....................................A...........T..................A.....................A.G..
ppy_6     ........................A.....................................A...........T........................................A.G..
ppy_7     ..............................................................A...........T........................................A.G..
ppy_8     ..............................................................A...........T........................................A.G..
ppy_17    ...............G..............................................A....................................................A.G..
ppy_18    ...............G.................................A............A....................................................A.G..
ppy_19    ...................................A..........................A....................................................A.G..
ppy_20    ..............................................................A....................................................A.G..
ppy_20a   ..............................................................A....................................................A.G..
ppy_21    ................................................A.............A....................................................A.G..
ppy_22    ..............................................................A.....................T..............................A.G..
ppy_23    ..............................................................A....................................................A.-..
ppy_24    ..............................................................A....................................................A.-..
ppy_25    ..............................................................A....................................................A.-..
ppy_26    ..............................................................A....................................................A.G..
ppy_28    ..............................................................A....................................................A.G..
ppy_29    ...............G..............................................A....................................................A.G..
ppy_30    ...............G..............................................A....................................................A.G..
ppy_31    ...............G..............................................A....................................................A.G..
```

```
Outgroup  TTGTAAAAATGATCAATAAATTGAATCAACAACACACCAGGAACCTTCCTACCCCAGAATA
ppy_4     ...C.........G.......A............T.A...ACG.......C.AA..C....
ppy_6     ...C.........G.......A............T.A...ACG.......C.AA..C....
ppy_7     ...C.........G......GA............T.A...ATG.......C.AA..C....
ppy_8     ...C.........G......GA............T.A...ATG.......C.AA..C....
ppy_17    .........................................CC.......G..A.......
ppy_18    .........................................CC.......G..A.......
ppy_19    .........................................CC.......G..A.......
ppy_20    .........................................CC.......G..A.......
ppy_20a   .........................................CC.......G..A.......
ppy_21    ................................G........CC.......G..A.......
ppy_22    .........................................CC.......G..A.......
ppy_23    .........................................CC.......G..A.......
ppy_24    .........................................CC.......G..A.......
ppy_25    .........................................CC.......G..A.......
ppy_26    .........................................CC.......GA.A.......
ppy_28    .........................................CC.......G..A.......
ppy_29    ...............................---.......CC.......G..A.......
ppy_30    ...............................---.......CC.......G..A.......
ppy_31    ...............................---.......CC.......G..A.......
```

Supplementary figure S2: Maximum likelihood phylogenetic trees of *NPIP* exon sequences. The trees are rooted on the respective rhesus *NPIP* sequences. Trees were generated in MEGA X (Kimura 2-parameter; n = 10000 bootstrap replicates). The approximate position of the sequences used in the analysis is indicated by orange boxes. 3'TD - 3' transduction

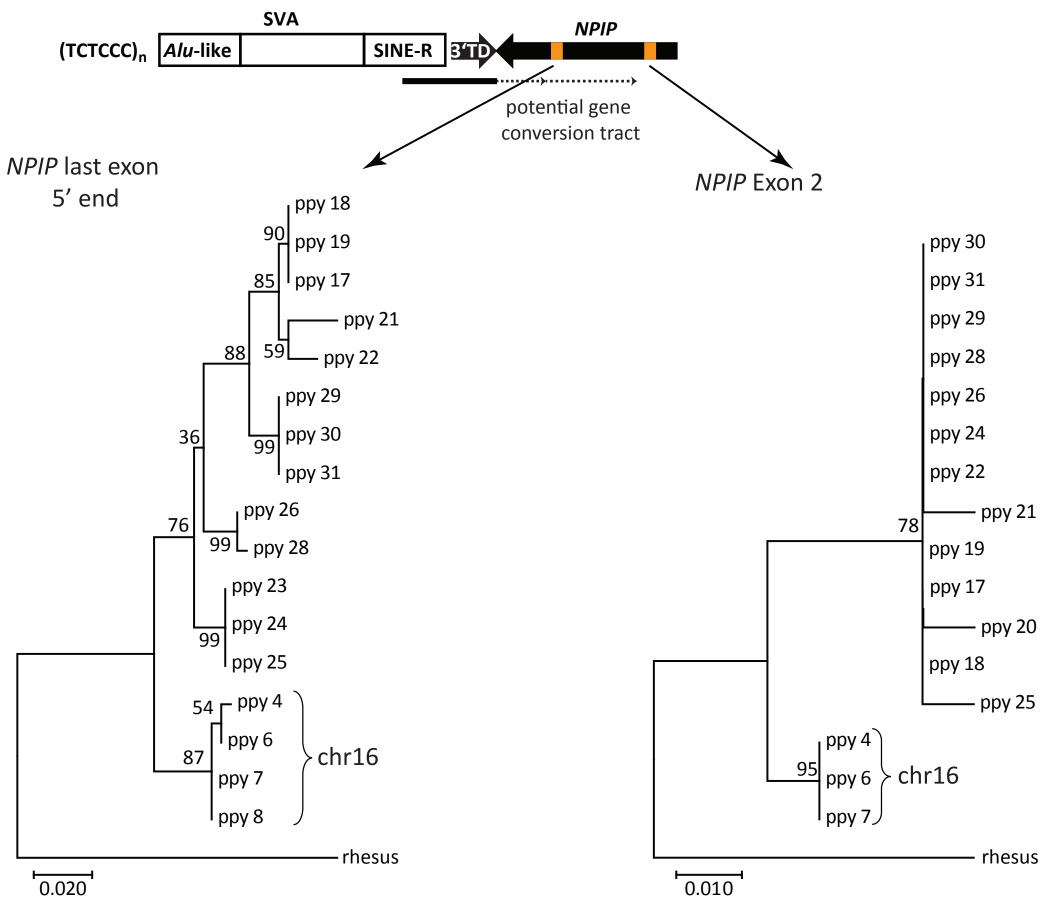

Supplementary figure S3: Multiple alignment of the chimeric SVAs (5' D/B and 3' B/D) resulting from integration of the lcr16a circular intermediate. Sequences of the 5' and 3' SVAs are those present in AC277974. SVA_D and SVA_B represent the Repeatmasker consensus sequences of the subfamilies. The VNTR was excluded from the alignment. The border between *Alu*-like region and SINE-R is marked with N and highlighted in yellow. Diagnostic subfamily-specific substitutions found in the chimeric elements are highlighted in blue.

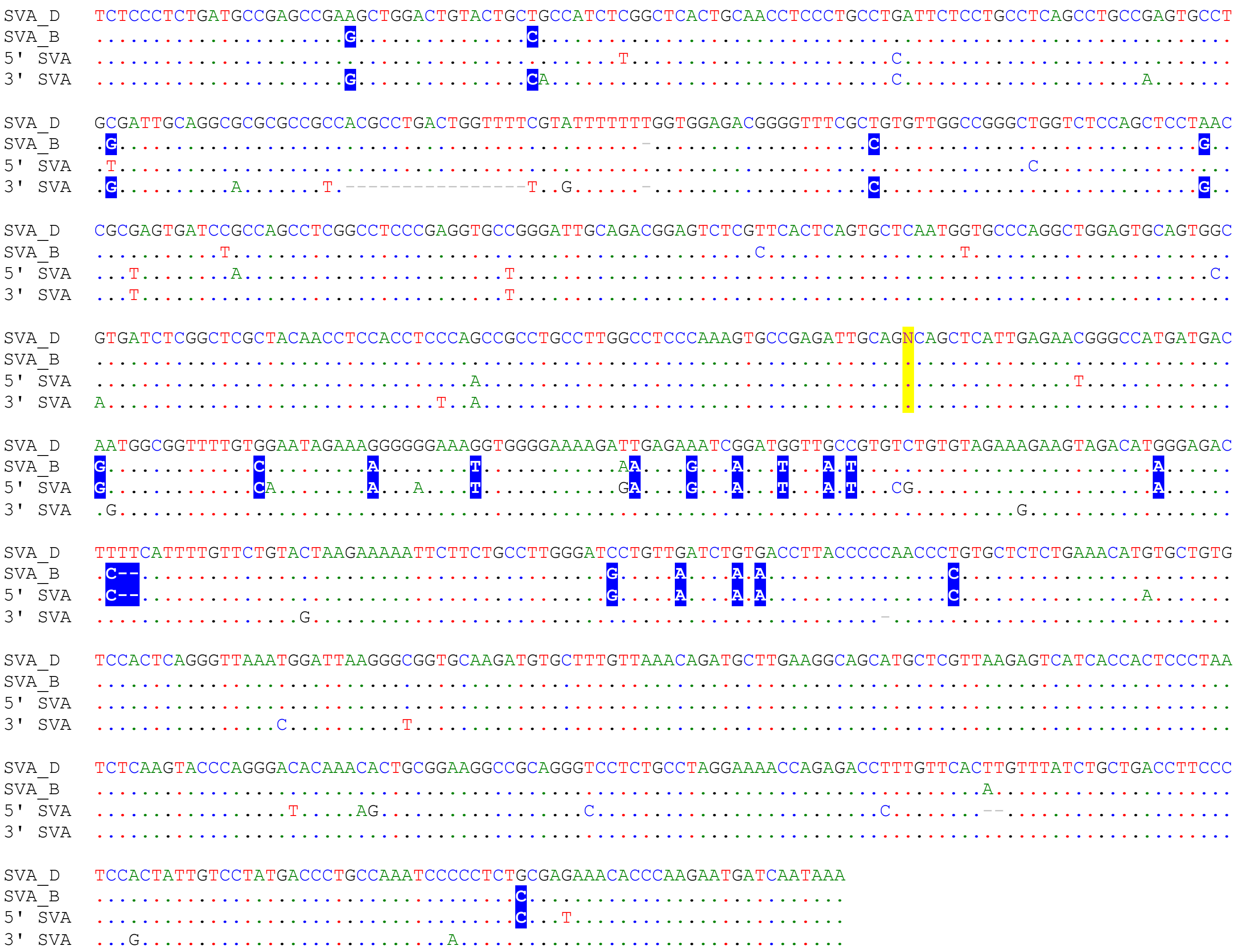

Supplement: msac103_Supplementary_Data [file msac103_supplementary_data.zip › Supplementary figures_DAMERT.pdf]
